# Removal of bovine digital dermatitis-associated treponemes from hoof knives after foot-trimming: a disinfection field study

**DOI:** 10.1186/s12917-020-02552-8

**Published:** 2020-09-11

**Authors:** A. V. Gillespie, S. D. Carter, R. W. Blowey, G. J. Staton, N. J. Evans

**Affiliations:** 1 Department of Infection Biology, Institute of Infection and Global Health, University of Liverpool, Merseyside, UK; 2 Wood Veterinary Group, 125 Bristol Road, Gloucester, GL2 4NB UK

**Keywords:** Dairy cattle, Digital dermatitis, Bovine, Treponemes, Disinfection, Hoof knives, Foot-trimming

## Abstract

**Background:**

Bovine digital dermatitis (BDD) is an infectious foot disease found commonly in dairy herds. Foot-trimming is an important husbandry procedure for reducing the ensuing lameness; however, epidemiological, and microbiological studies have identified this as a risk activity for transmitting BDD.

Three disinfectants have previously been identified in laboratory work as effective for removing viable BDD-associated *Treponema* spp., from hoof knife blades. The present study enrolled 133 dairy cattle with BDD lesions, and swabbed hoof knife blades before and after foot-trimming, and after knife disinfection with one of three disinfectants (1:100 FAM30®, 2% Virkon® and 2% sodium hypochlorite) to assess their efficacy under field conditions.

**Results:**

Detection of BDD treponeme phylogroup DNA was undertaken by direct PCR of swabs, and viable treponemes were detected by PCR of swab cultures after 6 weeks’ incubation.

Where hoof knives did not contact the lesion, BDD-associated treponemes were detected after foot-trimming in 12/22 (54.5%) cases by direct PCR and 1/22 (4.5%) cases by PCR of cultured organisms. Where contact was made with the lesion, 111/111 (100%) samples taken after trimming were positive by direct PCR and 47/118 (39.8%) were positive by culture PCR. Viable organisms were identified in cultures from lesion stages M2, M3, M4 and M4.1. No viable organisms were detected after disinfection of hoof knives.

**Conclusions:**

Hoof knives post-trimming were frequently contaminated with BDD-associated treponeme DNA. Viable organisms were identified in cultures whether contact had been made between hoof knife and lesion or not, although contact clearly increased the frequency of detection of viable organisms. The three disinfectants tested were effective for removing viable organisms. The disinfection protocol used in this study should therefore be considered reliable for adoption as standard industry practice.

## Background

Bovine digital dermatitis (BDD) is an infectious foot disease found commonly in dairy herds. This important, painful cattle disease was first reported in 1974 in Italy [1] and has since been recognised globally [2–4]. Herd-level prevalence has been reported in the range 63.8–97.4%, indicating BDD is endemic in many countries [5–8]. Typically, BDD lesions are found affecting the skin between the heel bulbs. Their clinical appearance has been described and classified into five lesion stages, beginning with a small, focal active lesion (M1), through the larger active ulcerative stage (M2), to the healing stage (M3). Lesions often become chronic, forming hyperkeratotic scabs (M4), from which state they can ‘reactivate’ with small focal active lesions superimposed (M4.1) [9, 10].

Spirochaetal bacteria are consistently found in BDD lesions and are implicated as primary pathogens in the disease. Studies of BDD-associated spirochaetes, through sequence analysis of the 16S rRNA gene, identified several phylogroups of bacteria from the *Treponema* genus closely related to the human oral treponemes, *Treponema denticola* and *Treponema vincentii,* as well as the non-pathogenic *Treponema phagedenis,* originally isolated from the human urogenital tract [11, 12]*.* Subsequently, studies have identified three of these phylogroups consistently within BDD lesions and more detailed analyses have led to their classification as: *Treponema medium/ vincentii-like, Treponema phagedenis* and *Treponema pedis* [13–15]. These three diverse treponemal phylogroups are strongly associated with BDD lesions and are considered to work synergistically to increase disease severity [16].

There are several on-farm risk factors associated with the development of BDD; predominantly housing and environmental factors [17]. A substantial collective of literature has resulted in industry recommendations to concentrate on improving farm environmental hygiene to control BDD. The existing paradigm is that infection is spread predominantly from M2 and M4 lesions to healthy feet via the environment; however, treponemes have not been isolated in culture or detected by PCR from farm environments [18]. Hence, questions remain about the importance of slurry and the environment in disease transmission. Current industry advice regarding better slurry management may be beneficial as it may reduce the susceptibility of skin to invasion by treponemes, and effective footbathing protocols have generally proved useful in reducing case numbers; however, there are additional infection reservoirs which may be key to BDD control initiatives.

Epidemiological studies have previously identified the use of external foot-trimmers as a risk factor for higher numbers of BDD cases in affected herds [8, 19]. With dairy farm expansion and amalgamation across the last 50 years, use of external foot-trimmers operating high-throughput systems, where large numbers of cows are trimmed in succession, has increased. Previous work detected BDD-associated treponemes on hoof knives using PCR, raising concerns that these tools, which are moved quickly and frequently from foot-to-foot, may act as a fomite for BDD-associated treponemes and could be having an impact on case numbers in these systems [20]. BDD-associated *Treponema* have also been identified on hoof-trimming equipment using 16S rRNA gene sequencing [21]. It is not known, however, whether *Treponema* detected by PCR or 16S rRNA sequencing correspond to the presence of viable infectious organisms [20], and if they are viable, whether they remain at sufficient numbers to constitute an infectious dose. BDD treponemes are known to survive on hoof knife blades under aerobic laboratory conditions for up to 2 h suggesting they could be viable under field conditions and transferable between feet via hoof knives [22].

Laboratory work testing disinfectants against a *Treponema phagedenis*-like spirochaete to determine minimum inhibitory concentrations and minimum bactericidal concentrations showed a range of disinfectants to be effective even in the presence of 20% manure [23]. Subsequently, in laboratory bacterial challenge experiments, three disinfectants were identified as good candidates for hoof knife disinfection using a 20 s contact time [22]. The current study tests these three disinfectants; − 1:100 FAM30®, 2% Virkon® and 2% sodium hypochlorite, under field conditions during foot-trimming of dairy cattle.

## Results

**Fig. 1:**
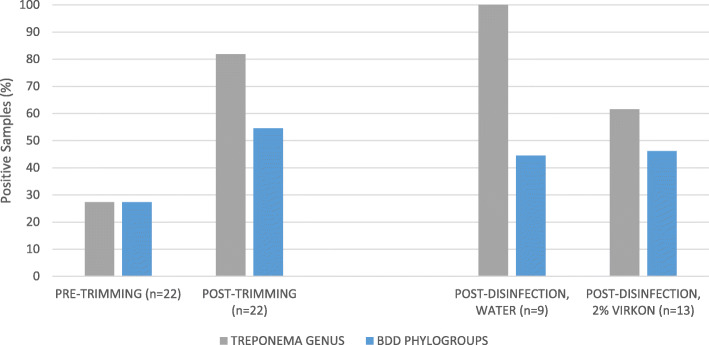
Direct PCR results showing disinfectant efficacy (no contact made between hoof knives and BDD lesions). *Treponema* DNA Positive Samples (%) identified by direct PCR showing efficacy of 2% Virkon® compared to water for disinfection of hoof knife blades for removal of *Treponema* genus DNA and DNA from three BDD-associated treponeme phylogroups. No contact was made between the hoof knife blades and the BDD lesions (*n* = 22)

**Fig. 2:**
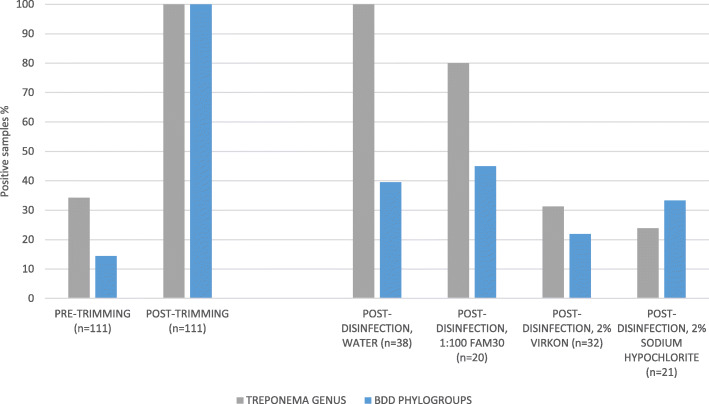
Direct PCR results showing disinfectant efficacy (contact made between hoof knives and BDD lesions). *Treponema* DNA Positive Samples (%) identified by direct PCR, showing efficacy of three disinfectants (compared to water) for disinfection of hoof knife blades for removal of *Treponema* genus DNA and DNA from three BDD-associated treponeme phylogroups. Contact was made between the hoof knife blades and the BDD lesions (*n* = 111)

A total of 133 BDD cases with the following pathological lesion stages were used to collect samples: one M1, 11 M2, 10 M3, 101 M4 and 10 M4.1 (Table 3). One of three disinfectants was used on trimming blades for 86 cases, whilst water was used in the remaining 47 cases. For 22 cases, no blade contact was made with the BDD lesion during foot-trimming (Fig. 1), whilst for the remaining 111 cases blade contact was made with the lesion (Fig. 2, Table 2), which was done to remove crusting from lesions prior to the application of topical treatment (Terramycin Aerosol Spray, Zoetis, UK).

Where contact was not made with BDD lesions, 18/22 (81.8%) of post-trimming swabs taken directly from the hoof knife blades tested positive (using nested PCR) for the *Treponema* genus, and 12/22 (54.5%) tested DNA positive for at least one of the three pathogenic Treponeme phylogroups after trimming (Fig. 1). After disinfection, 17/22 (77.3%) remained DNA positive for the *Treponema* genus and 10/22 (45.5%) remained DNA positive for at least one of the three pathogenic phylogroups. All knives cleaned with water- 9/9 (100%)- and 8/13 (61.5%) of those disinfected with 2% Virkon® remained DNA positive for the *Treponema* genus. The number of knives testing positive for BDD phylogroup DNA post-cleaning with water increased from 2/9 (22.2%) to 4/9 (44.4%), whilst disinfection using 2% Virkon® reduced the number of positive samples from 10/13 (76.9%) to 6/13 (46.2%) (Fig. 1). Some samples taken pre-trimming (6/22; 27.3%) tested DNA positive for *Treponema* genus and BDD phylogroups, suggesting some hoof knife blades were contaminated prior to trimming, including some that had not been previously used for hoof trimming that day.

Post swab culture (6 weeks), where no blade contact was made with BDD lesions, the *Treponema* genus and pathogenic treponeme DNA were detected in only 1/22 (4.5%) cases after trimming, indicating a low incidence of living organisms when sampled. Disinfection using 2% Virkon® removed culturable organisms in this case. No samples taken pre-trimming were found to contain culturable treponemes.

Where blade contact *was* made with BDD lesions (Fig. 2), all swabs (111/111) taken after trimming were DNA positive (using nested PCR) for the *Treponema* genus and at least one of the three pathogenic phylogroups. After disinfection, 69/111 (62.2%) remained PCR positive for the *Treponema* genus and 38/111 (34.2%) for at least one of the pathogenic phylogroups. Knives cleaned with water or disinfected with 1:100 FAM30® frequently remained contaminated with *Treponema* genus DNA (38/38 (100%) and 16/20 (75%) of samples respectively). They also performed less well for removing BDD phylogroup DNA; with 15/38 (39.5%) and 9/20 (45.0%) samples remaining positive (Fig. 2). Univariable logistic regression showed that when knives were disinfected using 2% sodium hypochlorite or 2% Virkon® (compared to water) they had decreased odds of testing PCR positive for BDD-associated phylogroup and/ or *Treponema* genus DNA (Table 1).

**Table 1:** Results of univariable logistic regression showing decreased odds of detection of *Treponema* genus and/ or BDD-associated phylogroup DNA where 2% sodium hypochlorite or 2% Virkon® were used to disinfect hoof knives compared to water

Disinfectant	Odds Ratio	95% Confidence Interval	***P***-value	Standard Error
*1:100 FAM30®*	−2.09	− 4.42- 0.24	0.078	1.19
*2% sodium hypochlorite*	−4.31	−6.48- -2.15	< 0.001	1.11
*2% Virkon®*	−4.23	−6.30- -2.16	< 0.001	1.06
*Baseline*	3.83	1.85–5.81		1.01

For swabbing of blades where lesion contact was made, as found for the samples where no contact was made between the hoof knives and the BDD lesions, some samples taken pre-trimming tested positive for the *Treponema* genus (38/111 (34.2%)) and BDD phylogroups (16/111 (14.4%)), indicating some hoof knife blades had become contaminated prior to trimming (Fig. 2). Positive pre-trimming samples came both from knives that were being used for the first time that day, and from knives that had already been used for foot-trimming during the session. However, Table 2 shows that these treponemes were not viable.

**Table 2:** *Treponema* PCR results for swab cultures showing disinfectant efficacy where contact was made between hoof knives and BDD lesions. The effect of disinfectants on viable treponemes on hoof trimming knives (determined by PCR of cultures), swabbed before use, and again post-trimming and post-disinfection. In all samples knife-BDD lesion contact occurred during trimming

Disinfectant	PRE-TRIMMING		POST-TRIMMING		POST-DISINFECTION	
	Treponema genus	BDD phylogroups	Treponema genus	BDD phylogroups	Treponema genus	BDD phylogroups
*2% Virkon® n* = 32	0/32	0/32	18/32 (56.3%)	13/32 (40.6%)	0/32	0/32
*2% sodium hypochlorite n* = 21	0/21	0/21	10/21 (47.6%)	10/21 (47.6%)	0/21	0/21
*1:100 FAM30® n* = 20	0/20	0/20	12/20 (60.0%)	10/20 (50.0%)	0/20	0/20
*Water n* = 38	0/38	0/38	24/38 (63.2%)	14/38 (36.8%)	0/38	0/38
*Total* (*n* = 111)	0/111	0/111	64/111 (54.2%)	47/111 (39.8%)	0/111	0/111

Where contact *was* made with BDD lesions (*n* = 111), the *Treponema* genus was detected by nested PCR of six-week cultures in 64/111 (57.7%) of cases and BDD-associated treponemes were detected in 47/111 (42.3%) of cases after trimming. Univariable logistic regression showed that making contact between the hoof knife blade and the lesion was statistically more likely to result in a positive culture (Odds Ratio 3.39, 95% confidence interval 1.35–5.43, *P* = 0.001). All three disinfectants (and water) were effective at removing culturable organisms (Table 2).

In an analysis of treponemes on blades used to trim different lesion stages, there was a clear presence at all M grades (Table 3). All BDD lesion stages showed detection of BDD treponemes in post-trimming cultures, except for a single M1 case in which there was no contact between the hoof knife blade and the lesion. Some lesion stages yielded higher proportions of positive cultures: 80, 75 and 66.7% from M2, 3 and 4.1 lesions respectively compared to 36.2% for chronic M4 cases; however, univariable logistic regression did not show these differences to be statistically significant.

**Table 3:** Detection of BDD treponeme phylogroups according to lesion stage. The effect of BDD lesion stage on detection of BDD treponeme phylogroups in post-trimming and post-disinfection hoof knife swab samples (disinfected using water, 2% Virkon®, 2% sodium hypochlorite or 1:100 FAM30®) as measured by both direct and post-culture PCR

Lesion Type	Contact with lesion (Yes/No)	Post-trimming Direct PCR	Post-disinfection Direct PCR	Post-trimming Culture PCR	Post-disinfection Culture PCR
M1(*n* = 1)	No	1/1 (100%)	1/1 (100%)	0	0
M2(*n* = 7)	No	3/7 (42.9%)	2/7 (28.6%)	0	0
M2(*n* = 5)	Yes	5/5 (100%)	2/5 (40.0%)	4/5 (80.0%)	0
M3(*n* = 6)	No	4/6 (66.7%)	3/6 (50.0%)	1/6 (16.7%)	0
M3(*n* = 4)	Yes	4/4 (100%)	3/4 (75.0%)	3/4 (75.0%)	0
M4(*n* = 7)	No	3/7 (42.9%)	3/7 (42.9%)	0	0
M4(*n* = 94)	Yes	94/94 (100%)	29/94 (40.4%)	34/94 (36.2%)	0
M4.1(*n* = 1)	No	1/1 (100%)	1/1 (100%)	0	0
M4.1(*n* = 9)	Yes	9/9 (100%)	4/9 (44.4%)	6/9 (66.7%)	0
All lesions (*n* = 22)	No	12/22 (54.5%)	10/22 (45.4%)	1/22 (4.5%)	0
All lesions (*n* = 111)	Yes	111/111 (100%)	38/111 (34.2%)	47/111 (39.8%)	0

A comprehensive list of cases showing both direct PCR and culture PCR results is available in Table 1 in Additional file 1.

## Discussion

This study has confirmed our previous work that the BDD-associated treponemes present in BDD lesions are readily transferrable to hoof trimming blades and can be viable and transmissible. Importantly, it has also shown, in field studies, that even brief disinfection of the blades (at shorter than typically recommended contact times) is very efficient in eliminating viable treponemes from blades and presumably blocking at least this route of BDD transmission. This is a very practical outcome worthy of consideration for best practice as it can be performed with minimal effort or investment.

In over 90% of samples, direct nested PCR testing detected post-trimming contamination of hoof knives with the *Treponema* genus DNA and at least one of the three culturable BDD-associated treponeme phylogroups. This is consistent with findings from previous field work [20] and the present study achieved similar overall disinfection efficacy for the BDD phylogroups as determined by the presence of BDD treponeme DNA within swab samples. However, detection of bacterial DNA direct from swabs does not assess the viability of organisms and therefore does not indicate whether they might be capable of transmission. For this reason, we introduced the use of bacterial culture of blade swabs, which showed that in 48/133 (36.1%) cases, post-trimming hoof knives were contaminated with BDD phylogroup treponemes that were viable. This is surprisingly high considering that treponemes are notoriously fastidious [13] and therefore their survival during sample collection and transport under aerobic conditions (especially when they are considered to be anaerobic organisms) would be expected to be low [24]. In addition, field samples inevitably contain many contaminating bacteria that could be expected to out-compete treponemes in culture. For these reasons, those samples where treponeme DNA was detected but cultures were negative should not automatically be regarded as not containing viable treponemes. In particular, the lower probability of culturing treponemes where no knife contact was made with lesions was unsurprising as lesions are the major source of bacteria, and a reduction in the number of positive cultures could be expected due to reduced bacterial load.

It was confirmed, by univariable logistic regression, that contact with the BDD lesions during foot-trimming increases the frequency of culturable BDD-associated treponemes. Infection rates may also vary according to BDD prevalence on farm; furthermore, management measures taken to reduce environmental bacterial load are likely to reduce foot infections. For example, recently footbathed feet may have fewer viable treponemes, which would result in a reduction in new BDD cases observed [25].

In this study, water was equally as effective at removing viable treponemes from hoof knives as any of the three disinfectants tested, although the sample size may be too small to differentiate. Water was not successful during laboratory testing where the majority of knives remained contaminated with viable treponemes; however, the in vitro experiments were carried out using pure treponeme cultures and are likely to represent a greater bacterial load [22]. Nevertheless, in case of greater bacterial challenge on the farm than that encountered in this trial, water should still be considered inferior to the three disinfectants for hoof knife disinfection given the previous laboratory collected data and the potential for presence of other bacteria likely to contribute to BDD lesions.

FAM 30® was effective for removing viable treponemes but did not perform as well as the other disinfectants for removing DNA detectable by direct PCR. The same in vitro study found that FAM 30® failed to eliminate *Treponema phagedenis* or *Treponema pedis* DNA from hoof knife blades despite removing all viable bacteria [22]. Whilst the current study does not have the power to differentiate the ability of each disinfectant to remove culturable treponemes (since all were 100% effective), it does show statistically significant differences between disinfectants in terms of successful removal of DNA (Table 1). Interpretation of these results is difficult because, as already stated, PCR positive results do not necessarily correspond to viable bacteria that could be capable of transmission; DNA can still be detected even when all bacteria are completely inactivated.

Positive cultures came from cases with M2, M3, M4 and M4.1 lesions. It has been shown that treponeme numbers are higher in active ulcerative lesions (as previously determined using qPCR to quantify bacterial DNA, rather than using culture) [16]. Our results indicate that higher percentages of M2, M3 and M4.1 lesions lead to positive cultures post-trimming when compared to the chronic M4 stage; however, this effect could be due to smaller sample sizes in the other lesion categories and overall insufficient sample size. This distribution of BDD lesion stages in the field is consistent with a recent study that classified 63.4% of heel bulb lesions as M4 [26], and a recent study of BDD transmission dynamics which found that about 70% of infected time was spent as M4 [27].

Pre-trimming contamination of hoof knives was an unexpected finding. It is possible that washing between cows was inadequate, and treponeme DNA was robust enough to remain intact after cleaning and immersion in 70% ethanol for 10 min. This would not, on its own, be important for disease transmission (as no viable organisms were detected) but could explain our pre-trimming data sets. It is also possible that contamination was caused by aerosolisation of bacteria in the vicinity of the foot-trimming crush, contaminating knives as they were air dried for use. Although studies regarding bio-aerosols on dairy farms are limited, it has been shown that *Mycobacterium avium subsp. paratuberculosis* can be detected in settled dust particles inside dairy housing 3 weeks after introduction of infected cattle [28]. Furthermore, spirochaetes have been identified in aerosols on a dairy farm, representing 1% of the total 16S rRNA gene sequences identified in aerosol samples [29]. As pre-trimming contamination did not result in viable treponeme cultures, we consider aerosols have limited ability to transmit *Treponema* spp.

Coupled with previous findings that treponemes can survive on hoof knife blades for 2 h and subsequently be recovered in culture [22], the finding of viable treponemes post-trimming in field samples provides evidence that poor hygiene during hoof trimming is a risk factor for BDD transmission. BDD infection models have shown that existing tissue damage and direct contact with fresh lesional material containing a viable polytreponemal bacterial load is needed to cause lesion development [30–32]. Foot-trimming would appear to fulfil the criteria for the infection models as viable treponemes (and potentially other bacteria) may be transferred between the feet of cows in the herd trimmed in quick succession (assuming that effective disinfection is not practised), and these feet frequently demonstrate some evidence of damage either from slurry exposure or general mechanical damage. Consideration should also be given to other aspects of hygiene during foot-trimming, particularly cleanliness of gloves, since it has been shown that treponemes can survive on gloves for up to 3 days after handling sheep feet showing the analogous disease, contagious ovine digital dermatitis [24]. The use of disinfectants on trimming tools will not only assist in controlling transmission of treponemes between animals and farms but will also have the effect of reducing transmission of other microbes which are known to contribute to digital dermatitis and other important foot lesions [33].

## Conclusions

Previous epidemiological studies identified use of an external foot-trimmer and lack of washing of hoof trimming equipment as risk factors for increased herd BDD prevalence [8, 19]. The current study demonstrates high levels of pathogenic treponeme contamination on hoof-knives post-trimming, including viable organisms, even where no contact has been made between the hoof knife and the BDD lesion. Collectively, the evidence provides a compelling argument for improving hygiene during foot-trimming. The disinfectants used here have been shown to be effective against BDD treponemes on hoof knives both in the laboratory [22] and on farm during foot-trimming of dairy cows with a short contact time of 20 s for removing viable treponemes. The disinfection protocol used in this study should therefore be considered reliable for adoption as standard industry practice.

## Methods

### Sample collection

The study included lactating dairy cattle during routine foot-trimming in three commercial farms (Cheshire and Gloucestershire, UK) where cows were housed in cubicles. The case definition was any foot showing visible BDD lesions of any pathological classification (M1, M2, M3, M4 or M4.1) [10]. Swab samples were taken during foot-trimming if a foot fitted the case definition. The hoof knives used belonged to the foot-trimmers who were participating in the study (Aesculap VC316V or VC300/ VC305, Neogen, USA). All foot-trimming was carried out according to the foot-trimmer’s normal protocol. Initial studies were made where the knife blades either did or did not come into contact with BDD lesions during trimming. However, swabs of blades which did not make lesion contact had a very low culture rate of pathogenic treponemes meaning that that efficacy of disinfectants could not be assessed for this group. Consequently, the approach was revised so that in subsequent studies only cases where hoof knife contact had been made with a BDD lesion to remove crusting from lesions prior to the application of topical treatment (Terramycin Aerosol Spray, Zoetis, UK) were included.

**Fig. 3:**
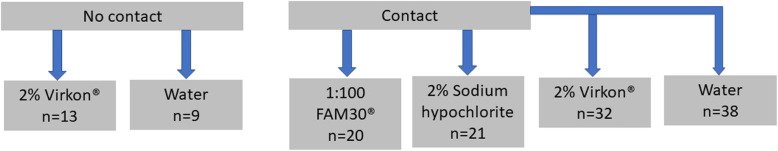
Experimental design of disinfection study. Shows the number of samples cleaned using each agent according to whether or not contact was made between the hoof knife blade and the BDD lesion for treatment purposes

At the beginning of each sample collection session and after each foot during foot-trimming, hoof knives were cleaned using household detergent in water (Fairy, Proctor & Gamble, USA) then immersed in 70% ethanol for a minimum of 10 min and air-dried prior to use. Plain cotton swabs (Copan, Italy) were passed back and forth three times over the whole length of the front and back of hoof knife blades to serve as pre-trimming control samples. Three swabs were taken per blade: one for inoculation into Oral Treponeme Enrichment Broth (OTEB, Anaerobe systems, USA) containing 10% heat-inactivated Foetal Calf Serum (FCS, Thermo Fisher Scientfic, USA), one for inoculation into OTEB containing 10% heat- inactivated Rabbit Serum (RS, Firstlink, UK) and one for direct testing by nested PCR without prior culture. Swab samples from the knives were taken again once foot-trimming of each foot was completed. Knives were rinsed briefly in water (3 s) to remove gross contamination before immersing the blades in one of three disinfectants (2% Virkon®, 2% sodium hypochlorite, 1:100 FAM30®) or water (as a comparison) for 20 s. The short contact time was chosen as the aim was to evaluate a rapid disinfection protocol which could be used by foot-trimmers with minimal disruption to workflow eg. by using two knives alternatively with one in disinfectant. Following disinfection, blades were shaken dry and three swab samples taken for a third time. The number of blades disinfected with each agent, according to whether contact was made with the BDD lesion is shown in Fig. 3. The culture protocol was designed to favour growth of the three cultivable BDD treponeme phylogroups so that the effect of disinfection could be evaluated. Liquid medium containing FCS favours the growth of *T. phagedenis-* like and *T. pedis* strains, whilst liquid medium containing RS favours growth of *T. medium/ vincentii*- like strains.

### Sample processing

On farm knife swabs for culture were immediately inoculated into their designated medium (OTEB) in 2 ml screw top tubes and transported at ambient temperature. On return to the laboratory they were placed in an anaerobic cabinet (Don Whitley Scientific, UK) (85% N_2_, 10% H_2_ and 5% CO_2_, 36 °C) and rifampicin and enrofloxacin added to a final concentration of 5 μg/ml and 1 μg/ml respectively to suppress growth of contaminants. After 6 weeks in culture, bacterial genomic DNA (gDNA) was extracted from cultures using a Chelex resin (Biorad, USA) protocol according to manufacturer’s instructions. Briefly, samples were boiled with the Chelex in a water bath for 15 min, then centrifuged at 13,000 rpm for 10 min [34]. Resulting gDNA-containing supernatant was frozen at -20 °C.

Swabs for direct nested PCR analyses were placed on ice for transport and gDNA subsequently extracted using a DNeasy® minikit (Qiagen, UK) according to manufacturer’s instructions and stored at -20 °C.

### PCR assays

Detection of *Treponema* was undertaken via two methods: nested PCR of sample swabs taken directly from the hoof knife blades, and nested PCR of DNA extracted from cultures given 6 weeks to grow. The former method is very sensitive for detecting *Treponema* DNA and the latter provides a measure of treponemal viability determined by an ability to grow in liquid culture medium. Whilst in this study treponemes were not isolated and identified in pure culture, the continued presence of their DNA after 6 weeks was interpreted as evidence of viable growth, a measure previously shown to correlate with observation of live treponemes in culture using phase contrast microscopy [22, 24].

All gDNA samples were subjected to nested PCR assays to a) detect the *Treponema* genus, and to b) specifically detect each of the three cultivable BDD-associated treponeme phylogroups. These were carried out using previously described primers [35] and Firepol® Taq polymerase (Solis Biodyne, Estonia) according to manufacturer’s instructions. Reaction conditions were as previously described [35], with an initial step using universal 16SrRNA gene bacterial primers, followed by a *Treponema* genus or phylogroup-specific nested PCR step, resulting in products which are 300-500 bp elements of the 16S rRNA gene. All reactions were carried out in triplicate and validated using gDNA from each BDD treponeme phylogroup as positive controls and double distilled water as negative controls [35].

PCR products were visualised after electrophoresis through 1% Agarose (Biorad, Hemel Hempstead, UK) and stained with 0.5 mg/ml ethidium bromide. PCR products were compared against 100 bp and 1 kb DNA ladders (ThermoFisher Scientific, Waltham, USA) for size determination. Electric current was supplied by Biorad Powerpac 300 (Biorad, Hemel Hempstead, UK) at 120 V, 400 mA for 45 min. Following electrophoresis, gels were transferred to a UV Illuminator, and images visualised and captured using a Geldoc gel documentation instrument and the GeneSys computer program (ThermoFisher Scientific).

### Statistical analyses

Univariable logistic regression was performed using STATA v14 (Statacorp, USA) to test whether contact between the hoof knife blade and the lesion, or the lesion stage, explained the outcome of post-trimming cultures. Each disinfectant used was tested as an explanatory variable for the outcome of detection of BDD-associated phylogroup and/ or *Treponema* genus DNA post-disinfection.

## Supplementary information

**Additional file 1: Table 1.**. Detection of the Treponema genus (T) and three BDD treponeme phylogroups (1, 2, 3) on 133 hoof knives during foot-trimming, using direct PCR of swabs and PCR of gDNA extracted from swab samples cultured for 6 weeks under anaerobic conditions.

## Data Availability

All data relevant to the study are included in the manuscript and supplementary information.
