# Supplementary material for: Removal of bovine digital dermatitis-associated treponemes from hoof knives after foot-trimming: a disinfection field study
Source: BMC Vet Res. 2020 Sep 11;16:330. doi: 10.1186/s12917-020-02552-8 (PMC7488572; doi:10.1186/s12917-020-02552-8)
Supplement: Supplementary file 1 — Additional file 1: Table 1.. Detection of the Treponema genus (T) and three BDD treponeme phylogroups (1, 2, 3) on 133 hoof knives during foot-trimming, using direct PCR of swabs and PCR of gDNA extracted from swab samples cultured for 6 weeks under anaerobic conditions. [file 12917_2020_2552_MOESM1_ESM.docx]

| **Table 1: Detection of the Treponema genus (T) and three BDD treponeme phylogroups (1, 2, 3) on 133 hoof knives during foot-trimming, using direct PCR of swabs and PCR of gDNA extracted from samples cultured for six weeks under anaerobic conditions.** | | | | | | | | | | | | | | | | | | | | | | | | | | | | |
| --- | --- | --- | --- | --- | --- | --- | --- | --- | --- | --- | --- | --- | --- | --- | --- | --- | --- | --- | --- | --- | --- | --- | --- | --- | --- | --- | --- | --- |
| FARM ID | DISINFECTANT | LESION CLASSIFI-CATION | CONTACT WITH LESION, YES OR NO | DIRECT PCR SWABS | | | | | | | | | | | | CULTURE RESULTS | | | | | | | | | | | | |
|  |  |  |  | PRE-TRIM | | | | POST-TRIM | | | | POST-DISINFECTION | | | | PRE-TRIM | | | | | POST-TRIM | | | | POST-DISINFECTION | | | |
|  |  |  |  | T | 1 | 2 | 3 | T | 1 | 2 | 3 | T | 1 | 2 | 3 | T | 1 | 2 | 3 | T | | 1 | 2 | 3 | T | 1 | 2 | 3 |
|  |  |  |  |  |  |  |  |  |  |  |  |  |  |  |  |  |  |  |  |  | |  |  |  |  |  |  |  |
| 1 | water | 1 | N | - | - | - | - | + | - | + | - | + | - | + | - | - | - | - | - | - | | - | - | - | - | - | - | - |
| 1 | water | 4.1 | N | - | - | - | - | + | - | + | - | + | - | + | - | - | - | - | - | - | | - | - | - | - | - | - | - |
| 1 | water | 3 | N | - | - | - | - | + | - | - | - | + | - | + | - | - | - | - | - | - | | - | - | - | - | - | - | - |
| 1 | water | 2 | N | + | - | - | - | + | - | - | - | + | - | + | - | - | - | - | - | - | | - | - | - | - | - | - | - |
| 1 | water | 2 | N | + | - | - | - | + | - | - | - | + | - | - | - | - | - | - | - | - | | - | - | - | - | - | - | - |
| 1 | 2% Virkon | 2 | N | - | - | + | - | - | - | + | + | - | - | - | - | - | - | - | - | - | | - | - | - | - | - | - | - |
| 1 | 2% Virkon | 4 | N | - | - | + | - | - | - | - | - | + | - | + | - | - | - | - | - | - | | - | - | - | - | - | - | - |
| 1 | 2% Virkon | 2 | N | - | - | - | - | - | - | + | - | + | + | + | - | - | - | - | - | - | | - | - | - | - | - | - | - |
| 1 | 2% Virkon | 3 | N | - | - | - | - | - | - | + | - | + | - | + | - | - | - | - | - | - | | - | - | - | - | - | - | - |
| 1 | water | 3 | N | - | - | - | - | + | - | - | - | + | - | - | - | - | - | - | - | - | | - | - | - | - | - | - | - |
| 1 | water | 2 | N | - | - | - | - | + | - | - | - | + | - | - | - | - | - | - | - | - | | - | - | - | - | - | - | - |
| 1 | water | 4 | N | - | - | - | - | + | - | - | - | + | - | - | - | - | - | - | - | - | | - | - | - | - | - | - | - |
| 1 | water | 4 | N | - | - | - | - | + | - | - | - | + | - | - | - | - | - | - | - | - | | - | - | - | - | - | - | - |
| 1 | 2% Virkon | 4 | N | - | - | - | - | + | - | - | - | + | - | - | - | - | - | - | - | - | | - | - | - | - | - | - | - |
| 1 | 2% Virkon | 3 | N | + | - | - | + | + | - | + | - | - | - | - | - | - | - | - | - | - | | - | - | - | - | - | - | - |
| 1 | 2% Virkon | 4 | N | - | - | - | - | + | + | + | - | - | - | - | - | - | - | - | - | - | | - | - | - | - | - | - | - |
| 1 | 2% Virkon | 3 | N | + | - | + | - | + | + | + | + | + | - | - | + | - | - | - | - | - | | - | - | - | - | - | - | - |
| 1 | 2% Virkon | 4 | N | - | - | - | - | + | + | + | + | + | - | + | + | - | - | - | - | - | | - | - | - | - | - | - | - |
| 1 | 2% Virkon | 3 | N | + | + | + | + | + | + | + | - | + | - | - | - | - | - | - | - | + | | - | + | - | - | - | - | - |
| 2 | 2% Virkon | 2 | N | - | - | - | - | + | - | - | - | - | - | - | - | - | - | - | - | - | | - | - | - | - | - | - | - |
| 2 | 2% Virkon | 2 | N | - | - | - | - | + | + | + | + | - | - | - | - | - | - | - | - | - | | - | - | - | - | - | - | - |
| 2 | 2% Virkon | 4 | N | + | - | + | + | + | - | + | + | + | - | + | + | - | - | - | - | - | | - | - | - | - | - | - | - |
| 3 | 2% sodium hypochlorite | 4 | Y | + | - | + | + | + | + | + | + | - | - | - | + | - | - | - | - | - | | - | - | - | - | - | - | - |
| 3 | 2% sodium hypochlorite | 4 | Y | + | - | + | + | + | + | + | + | + | - | - | + | - | - | - | - | - | | - | - | - | - | - | - | - |
| 3 | 2% sodium hypochlorite | 4 | Y | + | - | + | + | + | + | + | + | - | - | - | - | - | - | - | - | - | | - | - | - | - | - | - | - |
| 3 | 2% sodium hypochlorite | 4 | Y | - | - | - | - | + | + | + | + | + | - | + | + | - | - | - | - | - | | - | - | - | - | - | - | - |
| 3 | 2% sodium hypochlorite | 4 | Y | + | - | + | - | + | + | + | + | + | - | - | + | - | - | - | - | - | | - | - | - | - | - | - | - |
| 3 | 2% sodium hypochlorite | 4 | Y | - | - | - | - | + | + | + | + | - | - | - | - | - | - | - | - | - | | - | - | - | - | - | - | - |
| 3 | 2% sodium hypochlorite | 4 | Y | - | - | - | - | + | + | + | + | + | - | - | + | - | - | - | - | - | | - | - | - | - | - | - | - |
| 3 | 2% sodium hypochlorite | 4 | Y | - | - | - | - | + | + | + | + | - | - | - | - | - | - | - | - | - | | - | - | - | - | - | - | - |
| 3 | 2% sodium hypochlorite | 4 | Y | - | - | - | - | + | + | + | + | - | - | - | - | - | - | - | - | - | | - | - | - | - | - | - | - |
| 3 | 2% sodium hypochlorite | 4 | Y | - | - | - | - | + | + | + | + | - | - | - | - | - | - | - | - | - | | - | - | - | - | - | - | - |
| 3 | water | 4 | Y | - | - | - | - | + | + | + | + | + | - | - | - | - | - | - | - | - | | - | - | - | - | - | - | - |
| 3 | water | 4 | Y | - | - | - | - | + | + | + | + | + | - | - | - | - | - | - | - | - | | - | - | - | - | - | - | - |
| 3 | water | 4 | Y | + | - | - | - | + | + | + | + | + | - | - | - | - | - | - | - | - | | - | - | - | - | - | - | - |
| 3 | water | 4 | Y | + | - | - | - | + | - | + | + | + | - | - | - | - | - | - | - | - | | - | - | - | - | - | - | - |
| 3 | 2% Virkon | 4 | Y | + | - | - | - | + | + | + | + | + | + | + | + | - | - | - | - | + | | + | + | - | - | - | - | - |
| 3 | 2% Virkon | 4 | Y | - | - | - | - | + | + | + | + | + | - | - | - | - | - | - | - | - | | - | - | - | - | - | - | - |
| 3 | 2% Virkon | 4 | Y | + | - | - | - | + | + | + | + | - | - | - | - | - | - | - | - | - | | - | - | - | - | - | - | - |
| 3 | 2% sodium hypochlorite | 4 | Y | - | - | - | - | + | + | + | + | - | - | - | - | - | - | - | - | - | | - | - | - | - | - | - | - |
| 3 | 2% sodium hypochlorite | 4 | Y | - | - | - | - | + | + | + | + | - | - | - | - | - | - | - | - | + | | + | + | + | - | - | - | - |
| 3 | 2% sodium hypochlorite | 4 | Y | - | - | + | - | + | + | + | + | - | - | + | - | - | - | - | - | + | | - | + | - | - | - | - | - |
| 3 | 2% sodium hypochlorite | 4 | Y | - | - | - | - | + | + | + | + | - | - | - | - | - | - | - | - | + | | - | + | + | - | - | - | - |
| 3 | 2% sodium hypochlorite | 4 | Y | - | - | - | - | + | + | + | + | - | - | - | - | - | - | - | - | + | | + | + | + | - | - | - | - |
| 3 | 2% sodium hypochlorite | 4 | Y | - | - | - | - | + | + | + | + | - | - | - | - | - | - | - | - | + | | + | - | - | - | - | - | - |
| 3 | 2% sodium hypochlorite | 2 | Y | + | - | + | - | + | + | + | + | - | - | - | - | - | - | - | - | + | | + | + | - | - | - | - | - |
| 3 | 2% sodium hypochlorite | 4 | Y | + | - | - | - | + | + | + | + | - | - | - | - | - | - | - | - | + | | + | - | - | - | - | - | - |
| 3 | 2% sodium hypochlorite | 4 | Y | - | - | - | - | + | + | + | + | - | - | + | - | - | - | - | - | + | | + | + | + | - | - | - | - |
| 3 | 2% sodium hypochlorite | 4 | Y | - | - | - | - | + | + | + | + | + | - | - | - | - | - | - | - | + | | - | + | + | - | - | - | - |
| 3 | 2% sodium hypochlorite | 4 | Y | - | - | - | - | + | + | + | + | - | - | - | - | - | - | - | - | + | | - | - | + | - | - | - | - |
| 3 | 2% Virkon | 4 | Y | - | - | - | - | + | + | + | + | + | - | + | - | - | - | - | - | - | | - | - | - | - | - | - | - |
| 3 | 2% Virkon | 4 | Y | - | - | - | - | + | + | + | + | - | - | - | - | - | - | - | - | + | | - | + | - | - | - | - | - |
| 3 | 2% Virkon | 4 | Y | - | - | - | - | + | + | + | + | - | - | - | - | - | - | - | - | - | | - | - | - | - | - | - | - |
| 3 | 2% Virkon | 4 | Y | - | - | - | - | + | + | + | + | - | - | - | - | - | - | - | - | - | | - | - | - | - | - | - | - |
| 3 | 2% Virkon | 4 | Y | - | - | - | - | + | + | + | + | - | - | - | - | - | - | - | - | + | | - | + | - | - | - | - | - |
| 3 | 2% Virkon | 4 | Y | - | - | - | - | + | - | + | + | - | - | - | - | - | - | - | - | + | | - | - | - | - | - | - | - |
| 3 | water | 4 | Y | - | - | - | - | + | + | + | + | + | - | - | - | - | - | - | - | - | | + | + | - | - | - | - | - |
| 3 | water | 4 | Y | + | - | - | - | + | + | + | + | + | - | - | - | - | - | - | - | - | | - | - | - | - | - | - | - |
| 3 | water | 4 | Y | - | + | - | - | + | + | + | + | + | + | + | + | - | - | - | - | - | | - | - | - | - | - | - | - |
| 3 | FAM | 4 | Y | - | - | - | - | + | - | + | + | - | - | - | - | - | - | - | - | + | | - | - | - | - | - | - | - |
| 3 | FAM | 4 | Y | - | - | - | - | + | + | + | + | + | - | - | - | - | - | - | - | - | | - | - | - | - | - | - | - |
| 3 | FAM | 4 | Y | - | - | - | - | + | + | + | + | + | - | - | + | - | - | - | - | - | | - | - | - | - | - | - | - |
| 3 | FAM | 4 | Y | - | - | - | - | + | + | + | + | + | - | - | - | - | - | - | - | - | | - | - | - | - | - | - | - |
| 3 | FAM | 4 | Y | - | - | - | - | + | + | + | + | + | - | - | - | - | - | - | - | + | | - | + | - | - | - | - | - |
| 3 | FAM | 4 | Y | - | - | - | - | + | + | + | + | - | - | - | - | - | - | - | - | - | | - | - | - | - | - | - | - |
| 3 | FAM | 4.1 | Y | - | - | - | - | + | + | + | + | + | - | - | - | - | - | - | - | + | | + | + | - | - | - | - | - |
| 3 | FAM | 4.1 | Y | - | - | - | - | + | + | + | + | + | - | - | + | - | - | - | - | + | | - | + | - | - | - | - | - |
| 3 | FAM | 4 | Y | - | - | - | - | + | + | + | + | - | - | - | - | - | - | - | - | + | | + | + | + | - | - | - | - |
| 3 | FAM | 4 | Y | - | - | - | - | + | + | + | + | + | - | - | - | - | - | - | - | - | | - | - | - | - | - | - | - |
| 3 | FAM | 4 | Y | - | - | - | - | + | + | + | + | + | + | + | + | - | - | - | - | - | | - | - | - | - | - | - | - |
| 3 | FAM | 4 | Y | - | - | - | - | + | + | + | + | + | + | + | + | - | - | - | - | + | | - | - | - | - | - | - | - |
| 3 | FAM | 4.1 | Y | - | - | - | - | + | + | + | + | + | + | + | + | - | - | - | - | + | | + | + | + | - | - | - | - |
| 3 | FAM | 4 | Y | - | - | - | - | + | + | + | + | + | - | + | + | - | - | - | - | + | | - | + | + | - | - | - | - |
| 3 | FAM | 4 | Y | - | - | - | - | + | + | + | + | + | + | + | + | - | - | - | - | + | | + | + | + | - | - | - | - |
| 3 | FAM | 4 | Y | - | - | - | - | + | + | + | + | + | - | + | + | - | - | - | - | - | | - | - | - | - | - | - | - |
| 3 | FAM | 4 | Y | + | - | - | + | + | + | + | + | + | - | - | - | - | - | - | - | + | | - | + | - | - | - | - | - |
| 3 | FAM | 4 | Y | + | - | - | - | + | + | + | + | + | - | - | - | - | - | - | - | - | | - | - | - | - | - | - | - |
| 3 | FAM | 2 | Y | - | - | - | - | + | + | + | + | + | + | + | + | - | - | - | - | + | | - | + | + | - | - | - | - |
| 3 | FAM | 4 | Y | - | - | - | - | + | + | + | + | + | + | - | + | - | - | - | - | + | | - | - | - | - | - | - | - |
| 3 | water | 4.1 | Y | - | - | - | - | + | + | + | + | + | - | - | - | - | - | - | - | + | | - | + | - | - | - | - | - |
| 3 | water | 4 | Y | + | - | - | - | + | + | + | + | + | - | - | + | - | - | - | - | + | | - | - | - | - | - | - | - |
| 3 | water | 4 | Y | - | - | - | - | + | + | + | + | + | + | + | - | - | - | - | - | + | | + | + | - | - | - | - | - |
| 3 | water | 4 | Y | + | - | - | - | + | + | + | + | + | - | + | + | - | - | - | - | + | | - | + | + | - | - | - | - |
| 3 | water | 4 | Y | - | - | - | - | + | + | + | + | + | - | - | - | - | - | - | - | + | | - | + | + | - | - | - | - |
| 3 | water | 4 | Y | + | - | + | - | + | + | + | + | + | + | + | + | - | - | - | - | - | | - | - | - | - | - | - | - |
| 3 | water | 4 | Y | - | + | - | - | + | + | + | + | + | - | - | - | - | - | - | - | - | | - | - | - | - | - | - | - |
| 3 | water | 4 | Y | + | - | - | - | + | + | + | + | + | - | - | - | - | - | - | - | + | | - | - | - | - | - | - | - |
| 3 | water | 2 | Y | - | - | - | - | + | + | + | + | + | + | - | - | - | - | - | - | + | | + | + | + | - | - | - | - |
| 3 | water | 3 | Y | - | - | - | - | + | + | + | + | + | - | + | - | - | - | - | - | + | | + | + | + | - | - | - | - |
| 3 | water | 4 | Y | - | - | - | - | + | + | + | + | + | - | + | + | - | - | - | - | + | | - | + | - | - | - | - | - |
| 3 | water | 4 | Y | - | - | - | - | + | - | + | - | + | - | - | - | - | - | - | - | + | | - | - | - | - | - | - | - |
| 3 | water | 4 | Y | + | - | - | - | + | + | + | + | + | - | + | + | - | - | - | - | - | | - | - | - | - | - | - | - |
| 3 | water | 4 | Y | + | - | - | - | + | + | + | + | + | - | - | - | - | - | - | - | - | | - | - | - | - | - | - | - |
| 3 | water | 4 | Y | + | - | - | - | + | + | + | + | + | - | - | - | - | - | - | - | - | | - | - | - | - | - | - | - |
| 3 | 2% Virkon | 4 | Y | + | - | - | - | + | + | + | + | + | - | + | - | - | - | - | - | + | | - | + | - | - | - | - | - |
| 3 | 2% Virkon | 4 | Y | - | - | - | - | + | + | + | + | + | - | + | + | - | - | - | - | - | | - | - | - | - | - | - | - |
| 3 | 2% Virkon | 4.1 | Y | - | - | - | - | + | + | + | + | - | - | - | - | - | - | - | - | - | | - | - | - | - | - | - | - |
| 3 | 2% Virkon | 4 | Y | - | - | - | - | + | + | + | + | - | - | - | - | - | - | - | - | + | | + | - | - | - | - | - | - |
| 3 | 2% Virkon | 3 | Y | - | - | - | - | + | + | + | + | + | + | + | + | - | - | - | - | - | | - | - | - | - | - | - | - |
| 3 | 2% Virkon | 2 | Y | - | - | - | - | + | + | + | + | - | - | - | - | - | - | - | - | + | | + | + | + | - | - | - | - |
| 3 | 2% Virkon | 2 | Y | - | - | - | - | + | + | + | + | - | - | - | - | - | - | - | - | - | | - | - | - | - | - | - | - |
| 3 | 2% Virkon | 4 | Y | + | - | - | - | + | + | + | + | - | - | - | - | - | - | - | - | + | | + | + | + | - | - | - | - |
| 3 | 2% Virkon | 4 | Y | + | + | + | + | + | + | + | + | - | - | - | - | - | - | - | - | + | | + | + | + | - | - | - | - |
| 3 | 2% Virkon | 4 | Y | + | - | - | - | + | - | + | + | - | - | - | - | - | - | - | - | + | | - | - | - | - | - | - | - |
| 3 | 2% Virkon | 4 | Y | + | - | - | - | + | + | + | + | - | - | - | - | - | - | - | - | - | | - | - | - | - | - | - | - |
| 3 | 2% Virkon | 4 | Y | - | - | - | - | + | + | + | + | + | - | - | - | - | - | - | - | + | | + | + | + | - | - | - | - |
| 3 | 2% Virkon | 4 | Y | - | - | - | - | + | + | + | + | - | - | - | - | - | - | - | - | - | | - | - | - | - | - | - | - |
| 3 | 2% Virkon | 4 | Y | + | - | - | - | + | + | + | - | - | - | - | - | - | - | - | - | - | | - | - | - | - | - | - | - |
| 3 | 2% Virkon | 3 | Y | + | + | - | - | + | + | + | + | - | - | - | - | - | - | - | - | + | | + | + | + | - | - | - | - |
| 3 | water | 4 | Y | - | - | - | - | + | + | + | + | + | - | - | - | - | - | - | - | - | | - | - | - | - | - | - | - |
| 3 | water | 3 | Y | + | - | - | - | + | + | + | + | + | - | + | + | - | - | - | - | + | | + | + | + | - | - | - | - |
| 3 | water | 4 | Y | + | - | - | + | + | - | + | + | - | - | - | - | - | - | - | - | - | | - | - | - | - | - | - | - |
| 3 | water | 4 | Y | - | - | - | - | + | + | + | + | + | - | - | - | - | - | - | - | + | | - | - | - | - | - | - | - |
| 3 | water | 4 | Y | + | - | - | + | + | + | + | + | + | + | + | + | - | - | - | - | + | | - | - | - | - | - | - | - |
| 3 | water | 4.1 | Y | - | - | - | - | + | + | + | + | + | - | + | + | - | - | - | - | + | | - | + | + | - | - | - | - |
| 3 | water | 4.1 | Y | - | - | - | - | + | + | + | + | + | - | - | - | - | - | - | - | + | | - | - | - | - | - | - | - |
| 3 | water | 4 | Y | - | - | - | - | + | + | + | + | + | - | - | - | - | - | - | - | + | | - | - | - | - | - | - | - |
| 3 | water | 4 | Y | - | - | - | - | + | - | + | + | + | - | - | - | - | - | - | - | + | | - | - | - | - | - | - | - |
| 3 | water | 4 | Y | - | - | - | - | + | + | + | + | + | - | - | - | - | - | - | - | + | | - | - | - | - | - | - | - |
| 3 | water | 4 | Y | + | - | - | - | + | - | + | + | + | - | - | - | - | - | - | - | + | | - | + | - | - | - | - | - |
| 3 | water | 4 | Y | - | - | - | - | + | + | + | + | + | - | - | - | - | - | - | - | + | | + | + | + | - | - | - | - |
| 3 | water | 4 | Y | + | - | + | + | + | - | + | + | + | - | - | - | - | - | - | - | + | | - | - | - | - | - | - | - |
| 3 | water | 4 | Y | - | - | - | - | + | - | + | + | + | - | - | - | - | - | - | - | + | | - | - | - | - | - | - | - |
| 3 | water | 4 | Y | - | - | - | - | + | + | + | + | + | - | + | - | - | - | - | - | + | | + | + | + | - | - | - | - |
| 3 | water | 4.1 | Y | - | - | - | - | + | + | + | + | + | - | - | + | - | - | - | - | + | | - | + | - | - | - | - | - |
| 3 | 2% Virkon | 4 | Y | + | - | - | + | + | + | + | + | + | + | + | + | - | - | - | - | + | | + | + | + | - | - | - | - |
| 3 | 2% Virkon | 4 | Y | + | - | - | - | + | + | + | + | + | - | - | + | - | - | - | - | + | | - | - | - | - | - | - | - |
| 3 | 2% Virkon | 2 | Y | - | - | - | - | + | + | + | + | + | - | - | - | - | - | - | - | + | | - | - | - | - | - | - | - |
| 3 | 2% Virkon | 3 | Y | + | - | - | - | + | + | + | + | - | - | - | - | - | - | - | - | + | | + | - | - | - | - | - | - |
| 3 | 2% Virkon | 4 | Y | + | - | - | - | + | - | + | + | - | - | - | - | - | - | - | - | - | | - | - | - | - | - | - | - |
| 3 | 2% Virkon | 4 | Y | + | - | - | - | + | + | + | + | - | - | - | - | - | - | - | - | + | | - | + | + | - | - | - | - |
| 3 | 2% Virkon | 4 | Y | - | - | - | - | + | + | + | + | - | - | - | - | - | - | - | - | + | | - | - | - | - | - | - | - |
| 3 | 2% Virkon | 4.1 | Y | - | - | - | - | + | + | + | + | - | - | - | - | - | - | - | - | - | | - | - | - | - | - | - | - |
